# Genome-wide analysis of methylation in rat fetal heart under hyperglycemia by methylation-dependent restriction site–associated DNA sequencing

**DOI:** 10.1371/journal.pone.0268117

**Published:** 2022-05-11

**Authors:** Rui Meng, Junxian Song, Lina Guan, Qian Li, Cuige Shi, Dongmei Su, Xu Ma

**Affiliations:** 1 Graduate School of Peking Union Medical College, Chinese Academy of Medical Sciences, Beijing, China; 2 Department of Genetics, National Research Institute for Family Planning, Health Department, Beijing, China; 3 Department of Cardiology, Peking University People’s Hospital, Beijing, China; Tokyo University of Agriculture, JAPAN

## Abstract

Diabetes mellitus causes an increased incidence of congenital heart malformations. However, the pathogenesis and potential epigenetic mechanism involved in this process are unclear. In this study, we used MethylRAD sequencing to compare changes in methylation levels in the genomic landscapes in the fetal heart in a rat model of hyperglycemia. Our results showed that methylation of CCGG/CCNGG sites were mostly enriched in intergenic regions, followed by intron, exon, upstream and the 5′ and 3′ untranslated regions. qRT-PCR results confirmed the MethylRAD sequencing findings, suggesting that abnormal CCGG/CCNGG methylation in the upstream region regulated gene expression. The differential methylation genes (DMGs) based on the CCGG and CCNGG sites in the upstream region were examined by Gene Ontology and Kyoto Encyclopedia of Genes and Genomes analysis. Gene Ontology indicated that the CCGG-based DMGs involved in biological process and function were mainly related to transcription and co-SMAD binding. The CCNGG-based DMGs were mainly related to transcription and cytokine-mediated signaling pathways. Kyoto Encyclopedia of Genes and Genomes analysis indicated that CCGG-based DMGs were mainly involved in the Wnt signaling and TGF-β signaling pathways. CCNGG-based DMGs were involved in the TNF signaling and apoptosis pathways. These genes may play dominant roles in cardiomyocyte apoptosis and heart disease and require further study. These genes may also serve as potential molecular targets or diagnostic biomarkers for heart malformations under hyperglycemia.

## Introduction

Diabetes mellitus is a common complication of pregnancy that results in an increased incidence of congenital malformations, including heart defects [1–3]. Despite the improvements in obstetric care and management of maternal hyperglycemia over the last few decades, the perinatal mortality and congenital abnormality rates are higher in pregnancies complicated by diabetes by several fold compared with rates in healthy pregnancies [4]. However, few studies have examined the molecular basis of the pathogenesis of congenital heart disease (CHD) in pregestational diabetes and the factors responsible for the high incidence of CHDs under hyperglycemia.

Maternal illnesses, which affect the fetal environment and can cause birth defects, are proposed to be mediated by epigenetic mechanisms, including posttranslational modifications of histones, DNA methylation, and noncoding RNAs [5, 6]. Research has indicated that changes in the methylation level of genes related to cardiac development is one of the mechanisms underlying the pathogenesis of CHD. In one study, the methylation level of the NOX5 gene promoter region was increased in 66.67% of fetuses with ventricular septal defects [7]. Another report showed that abnormal methylation and expression of VangL2 gene are closely related to the occurrence of tetralogy of Fallot [8]. These studies suggest that methylation level changes of CHD-related genes play a role in the pathogenesis of CHD.

MethylRAD sequencing analysis is a new and powerful tool derived from a high-throughput sequencing platform that allows for exploration of epigenetic mechanisms as well as DNA methylation modifications in complex diseases on a genome-wide scale [9, 10]. The aim of this study was to investigate whether maternal diabetes affects fetal heart development through mechanisms involving DNA methylation. We used Methylation-RAD sequencing analyses to screen new methylation sites and various methylation genes related to heart development in response to the hyperglycemic environment.

## Material and methods

### Establishment of the diabetic animal model

Female and male Sprague Dawley rats (10–11 weeks of age) were housed with free access to food and water at a mean±standard deviation (SD) constant temperature of 22±2°C,humidity of 55±5%, and a 12-h light/12-h dark cycle. This study was performed in strict accordance with the recommendations in the Guide for the Care and Use of Laboratory Animals of the National Institutes of Health. The experimental protocol was approved by the National Institutes of Health Guide for Care and Use of Laboratory Animals. Establishment of the diabetic animal model was performed as previously described [11, 12]. Briefly, female Sprague–Dawley female rats were bred with male rats overnight. Vaginal plugs were observed the following morning, which was considered embryonic day 0.5 (E0.5). The pregnant rats were injected intraperitoneally with STZ at a dosage of 50 mg/kg. Glucose levels in maternal blood were detected from a tail nick using a Freestyle glucometer every other day. Diabetes was indicated when blood glucose levels were equal to or greater than 16.7 mM. At E15.5, the pregnant rats were euthanized; the diabetes-exposed embryos were collected by Caesarean section and the embryonic heart was removed for analysis.

### DNA sample isolation, Methylation-RAD library construction and high-throughput sequencing

Genomic DNA was extracted from fetal heart tissues of the control and diabetic groups using the QIAamp DNA Mini Kit (Cat No. 51306) following the manufacturer’s protocol. The Methylation-RAD tag libraries were constructed in six individuals with two groups following the protocol from previous studies [13, 14]. The Methylation-RAD library was prepared by digesting 200 ng genomic DNA from each sample using 4 U of FspEI (NEB, USA) at 37°C for 4 h. To verify the effectiveness of digestion, 4 μl of digested DNA (~50 ng) was run on a 1% agarose gel. FspEI recognizes 5-methylcytosine in CmCGG and CmCWGG sites and generates a double-stranded DNA break on the 3′ side of the modified cytosine at N12/N16. Symmetrical DNA methylated sites are bidirectionally cleaved by FspEI to generate 32 bp fragments. Two adaptors were added to the digested DNAs by T4 DNA ligase, and the ligation products were amplified in 20 μl reactions by specific primers. The PCR products were purified using a MinElute PCR Purification Kit (Qiagen) and pooled for sequencing using the Illumina X-ten PE 150 sequencing platform. Sequencing was performed by Shanghai Oebiotech Co. Ltd (Shanghai, China).

### Quality control and data alignment

Base quality values were calculated using a Phred quality score (Q sanger = -10log10p). Input sequencing data before operation and computing were called raw reads. Raw reads were first subject to quality filtering and adaptor trimming. After the operation, the data, including adapter reads and low-quality sequences, were removed from raw reads as clean reads. The clean reads were used for subsequent analyses. The Methyl-RAD sequencing data for each sample were compared with the reference genome (reference genome download link: ftp://ftp.ensembl.org/pub/release-84/fasta/rattus_norvegicus/dna/Rattus_norvegicus.Rnor_6.0.dna.toplevel.fa.gz) using the SOAP software (ver. 2.21; the primary alignment parameters included: -M4–v2–r0) [15]. DNA methylation sites with a sequence depth of no less than 3 were judged to be reliable.

### Quantitation of methylation level

Because of the consistency of amplification efficiency for sequences with equal length, the methylation level of the sites (CCGG/CCNGG) can be quantified by the sequencing depth of the methylation tag. For Methylation-RAD-sequencing, the methylation level of each site (CCGG/CCNGG) was represented by reads per million analysis. Reads per million is the unit of the quantitative value of methylation level at the site. The distribution trend of the methylation sites from different regions including 2 kb upstream/downstream of the transcription start site (TSS), gene body, and 2 kb upstream/downstream of the transcription termination site (TTS) were analyzed by SNPeff software (version 4.3p) [16].

### Comparison of methylation levels between the two groups

The change in methylation level between the diabetic and control groups was assessed using edgeR software [17], which relies on the sequencing depth information of each site in the relative quantitative results of methylation. A *P-value* <0.05 and log2FC >1 were considered statistically significant.

### Gene annotation and enrichment analysis

The gene annotation information of rat was downloaded from the National Center for Biotechnology Information (NCBI) at ftp://ftp.ensembl.org/pub/release-84/gff3/rattus_norvegicus/Rattus_norvegicus.Rnor_6.0.84.gff3.gz. The untranslated regions (UTRs) of genes were calculated using the SnpEff tool based on the annotation information (version 4.3p) [16, 18]. The distributions of the methylated sites on various genomic sequences were calculated by BED tools (v2.25.0) [19]. Cluster analysis was used to analyze the changes in CCGG/CCNGG methylation levels between the case and control groups.Gene functions were examined using Gene Ontology (GO) and Kyoto Encyclopedia of Genes and Genomes (KEGG) function enrichment analysis of the gene where the differential methylation site was located. The number of genes included in each GO entry and KEGG pathway was counted and the significance of gene enrichment for each GO entry and KEGG pathway was calculated using the hypergeometric distribution test. GO entries with more than two corresponding genes greater in three categories were screened Differences were considered significant at P < 0.05.

### RNA extraction and qRT-PCR analysis

Total RNA was extracted from the rat fetal heart tissues using Trizol reagent (Life Technologies) according to the manufactures instructions. Reverse transcription (RT-PCR) was performed using the Access RT-PCR System (Promega). Quantitative PCR was performed with SYBR Green (TaKaRa) used as a double-stranded DNA-specific fluorescent dye Reactions were conducted with 1 μL RT-PCR cDNA, 0.5 μL each of forward and reverse primers (10 μmol/L), 8μL water and 10 μL SYBR Green. The samples were run by the StepOne real-time PCR machine (ABI, USA).β-actin was used to normalize the data. Primer pairs for β-actin, Smad3, Dhfr, Sumo3, Pdp1 Mt-atp6 were showed in S1 Table). qRT-PCR was performed in triplicate.

### Statistical analysis

The Student t-test and ANOVA analysis were used to calculate the statistical significance of the experimental data. The significance level was set as * p < 0.05, ** p < 0.01. Error bars denote standard deviations.

## Results

### Methylation-RAD of the samples

**Fig 1:**
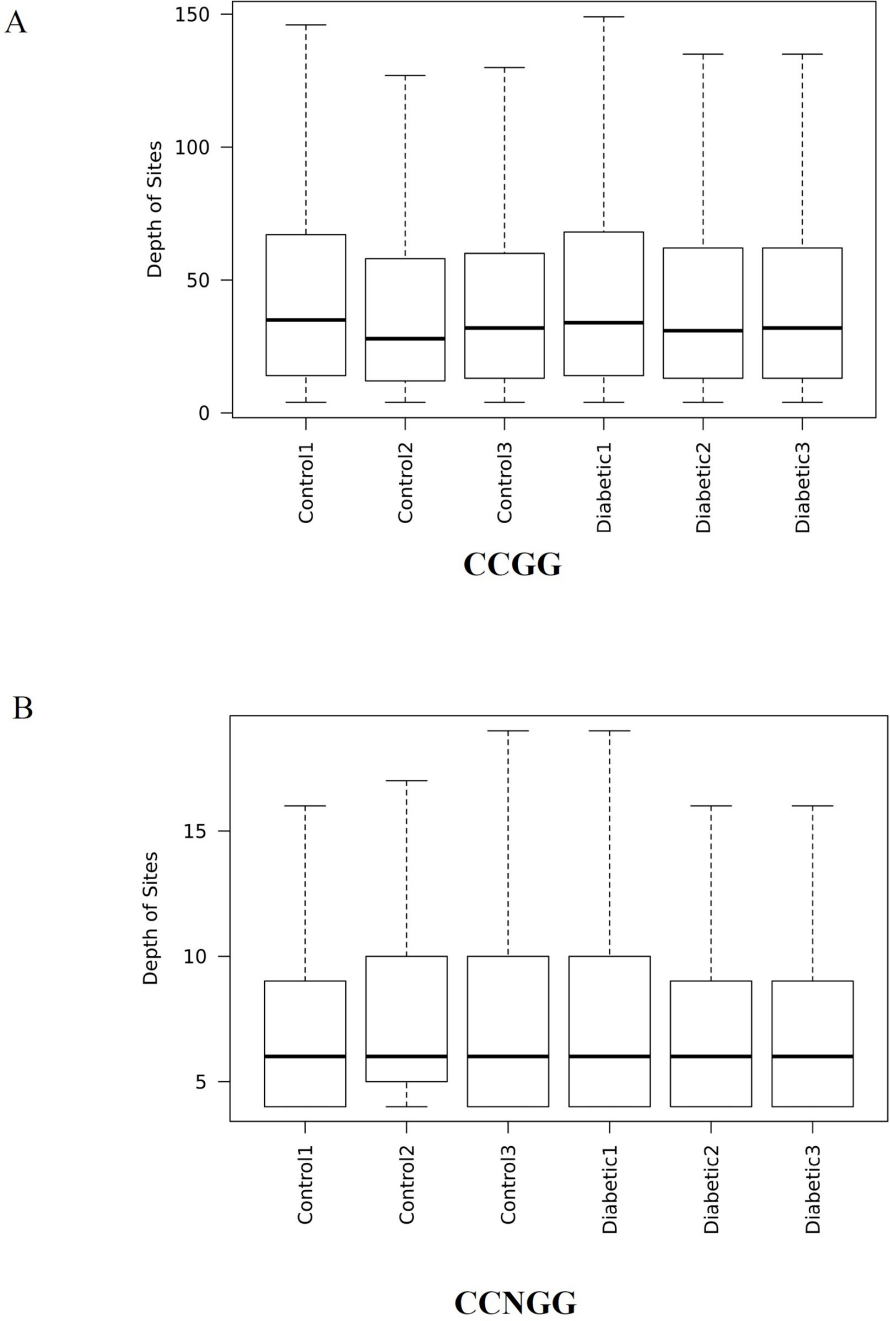
The sequencing depths of the DNA methylation sites (CCGG sites and CCNGG sites) in each sample are shown in a box plot. (A) Sequencing depth box diagram of CCGG methylation site in each sample. (B) Sequencing depth box diagram of CCNGG methylation site in each sample. In the box diagram, the box part is the main body of the box-shaped chart, and the middle of the black horizontal line is the median of the data.

We established diabetic rats as described in the Methods, and histological analysis revealed that fetal hearts from diabetic rats mainly showed cardiac developmental defects including chamber dilation, Myocardial tissue and cell reduction, and thinning of ventricular walls. These results were consistent with our previous studies [11, 12]. Atrial septal defect and ventricular septal defects are often observed in diabetes-exposed embryos with ventricular wall thinness (S1 Fig). To study the role of DNA methylation in embryonic heart development defects under hyperglycemia, we used Methylation-RAD sequencing to compare changes of the genomic methylation level in the fetal heart between the control and diabetic groups. As shown in Table 1, we obtained 113.99 million clean reads, on average, for each sample. After treatment with FspEI, on average, 49.96 million enzyme reads and 37.6 million mapping reads were generated. The mapped ratio in diabetic and control groups, on average, was approximately 75.24% (Table 1). The sequencing depths of the DNA methylation sites (CCGG sites and CCNGG sites) in each sample are shown in a box plot in Fig 1. As shown in Fig 1 and Table 1, the number of methylation sites in each sample had a depth higher than 3. Therefore, the sequencing reads satisfied the condition of the following analyses.

**Table 1:** Overview of sequencing and assembly.

Sample	Clean Reads	Enzyme Reads	Mapping Reads	Ratio
Control 1	112708549	54633878	41058688	75.15%
Control 2	112708549	46537797	34702028	74.57%
Control 3	112708549	47890930	35866154	74.89%
Diabetic 1	116547486	52753210	40435388	76.65%
Diabetic 2	112708549	48058471	36163648	75.25%
Diabetic 2	116547486	49860970	37359972	74.93%

### Distribution of DNA methylation sites in different functional regions

**Fig 2:**
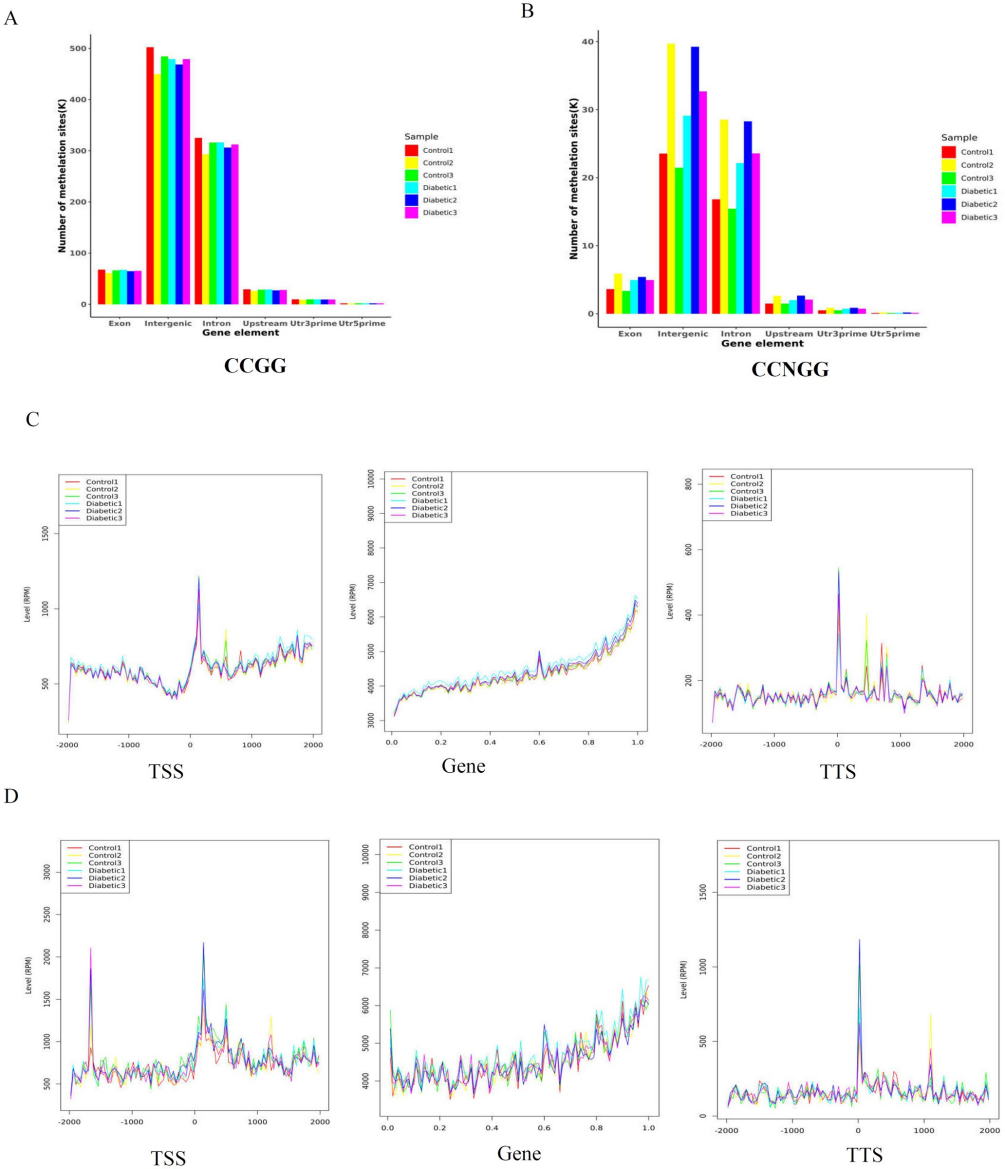
Distribution of DNA methylation sites in different functional regions. (A) CCGG methylation sites on different gene function components distribution histogram (B) CCNGG methylation sites on different gene function components distribution histogram. The y-axis shows the number of methylation sites. The x-axis shows the different components of the genome. (C) Distribution of CCGG methylation sites around gene bodies. The x-axis indicates the position around gene bodies, and the y-axis indicates the normalized read number. (D)Distribution of CCNGG methylation sites around gene bodies.

We next analyzed the distribution of the methylated CCGG/CCNGG sites in distinct genomic sequences, including 5′UTR, 3′ UTR, upstream, exon, intron, and intergenic regions (Fig 2A and 2B). Most DNA methylation sites were enriched in intergenic and intron regions, followed by exon and upstream regions; the proportion of methylation sites was the lowest in the 5′UTR and 3′ UTR. We further examined the distribution curves of DNA methylation in the 2 kb region upstream of the TSS, gene body, and the TTS. The region around the TSS is crucial for gene expression regulation. As shown in Fig 2C and 2D, the DNA methylation levels at CCGG or CCNGG sites in the gene body and TTS regions were similar between the two groups. The level of DNA methylation was low at 2 kb upstream of the TSS and increased significantly at the TSS; the methylation level then increased steadily throughout the gene body and began to decrease at the TTS. The DNA methylation distribution at the CCGG sites was different from the DNA methylation distribution at the CCNGG sites in the TSS region. The level of DNA methylation at the CCNGG site was lower 2 kb upstream of the TSS and increased suddenly near the TSS (left panel in Fig 2C). There were two peaks of increased level of DNA methylation at the CCNGG sites (left panel in Fig 2D).

### Comparison of methylation levels between the control and diabetic groups

**Fig 3:**
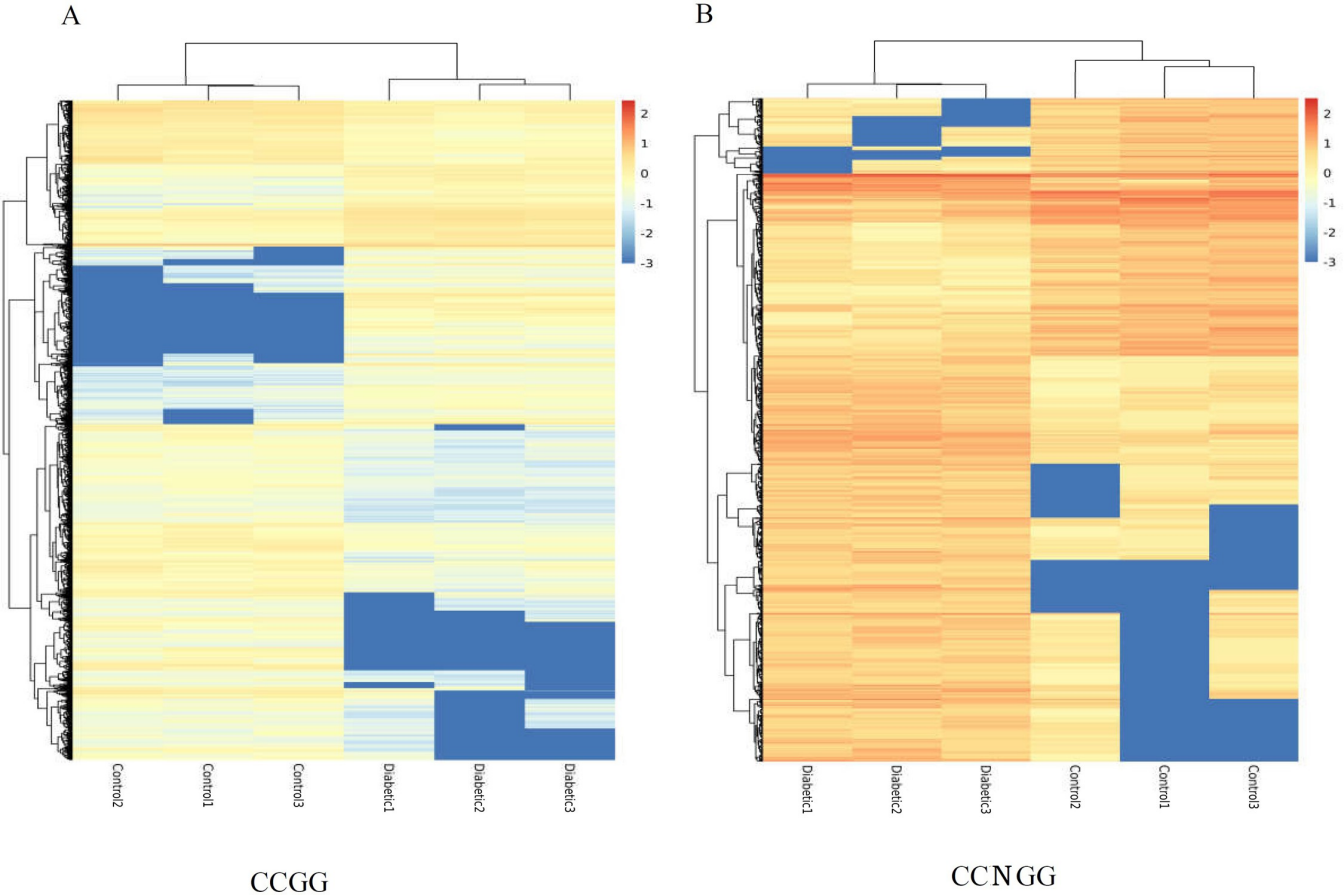
Comparison of methylation levels between the control and diabetic groups. (A) Cluster heat map of differentially CCGG sites methylation levels between the two groups. (B) Cluster heat map of differentially CNCGG sites methylation levels between the two groups.

We used cluster analysis to further reveal the changes in CCGG/CCNGG methylation levels between the diabetic and control groups for three biological replicates. As shown in Fig 3, there were different methylation patterns between the two groups. Hypomethylated CCGG sites in the diabetic group are clustered near the bottom, whereas hypomethylated CCGG sites in the control group are clustered in the upper-middle part (Fig 3A). Hypomethylated CCNGG sites in the diabetic group are clustered near the bottom, whereas hypomethylated CCGG/CCNGG in controls are clustered near the top (Fig 3B). These results revealed differences in hypomethylation of CCGG/CCNGG sites between the control and diabetic groups.

### Distribution of differential methylation sites on genes of different functional components

**Fig 4:**
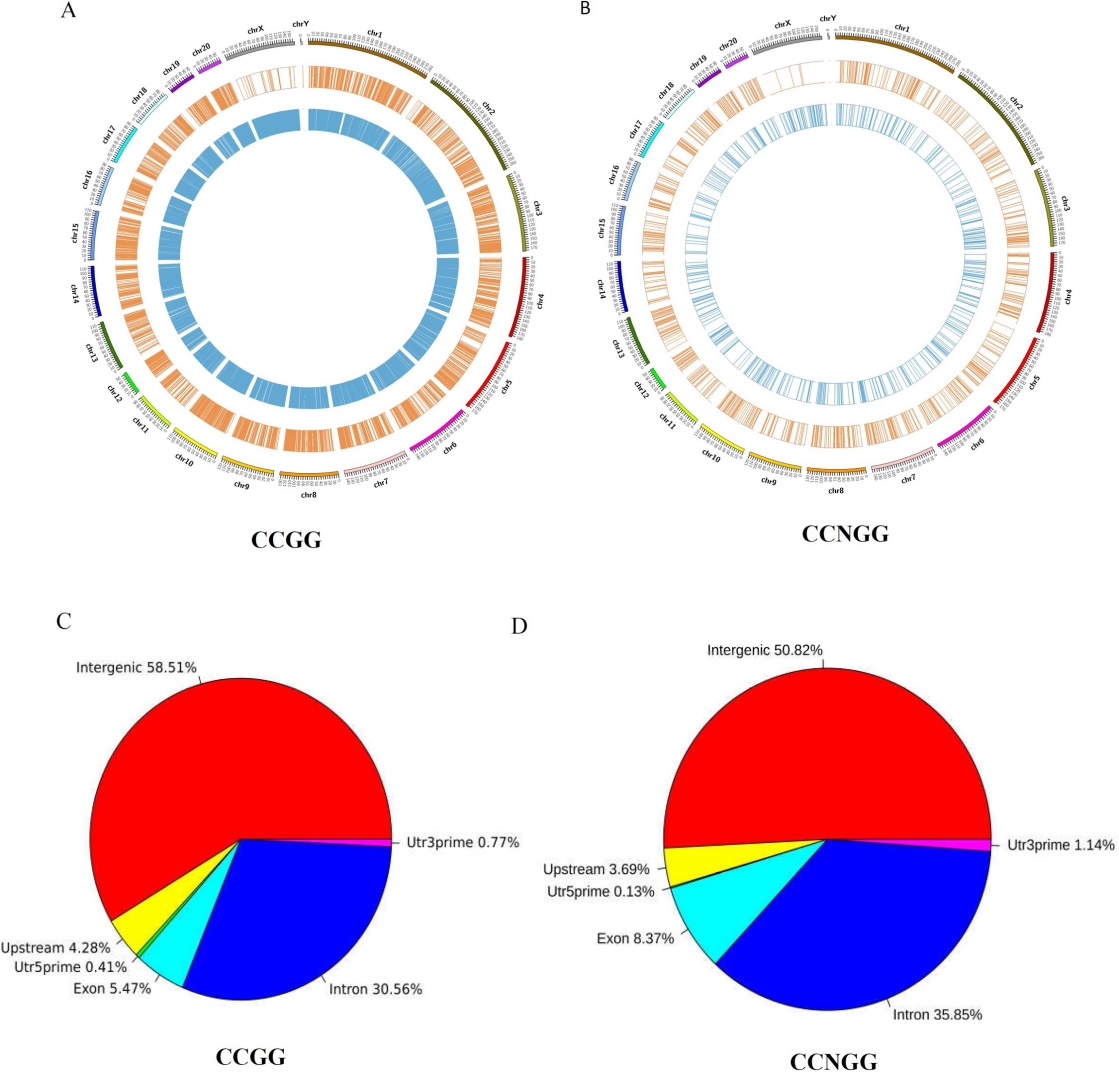
Distribution of differential methylation sites on genes of chromosomes and different functional components. (A) Distribution of differential CCGG methylation sites on chromosomes. (B) Distribution of differential methylation CCNGG sites on chromosomes. (C) Distribution of differential CCGG methylation sites on genes of different functional components. (D) Distribution of differential CCNGG methylation sites on genes of different functional components.

Following the positional information of the differentially methylated sites relative to the chromosome, the distribution Circos of differential methylation sites on chromosomes was drawn, and the results are shown in Fig 4A and 4B. The differential methylation sites on chromosomes of the brown track represent hypermethylated sites, whereas the blue track represented hypomethylated sites. The pie map distribution of differential methylation sites on different functional components was drawn following the positional information of the differentially methylated sites relative to the gene, and the results are shown in Fig 4C and 4D. The results showed that the differential CCGG/CCNGG methylation sites were mostly enriched in the intergenic regions, followed by the gene intron regions, the exon regions, and the upstream regions and finally the 5′UTR and 3′ UTR.

### Genes with abnormal methylation in the upstream region differentially expressed in the diabetic and control groups

**Fig 5:**
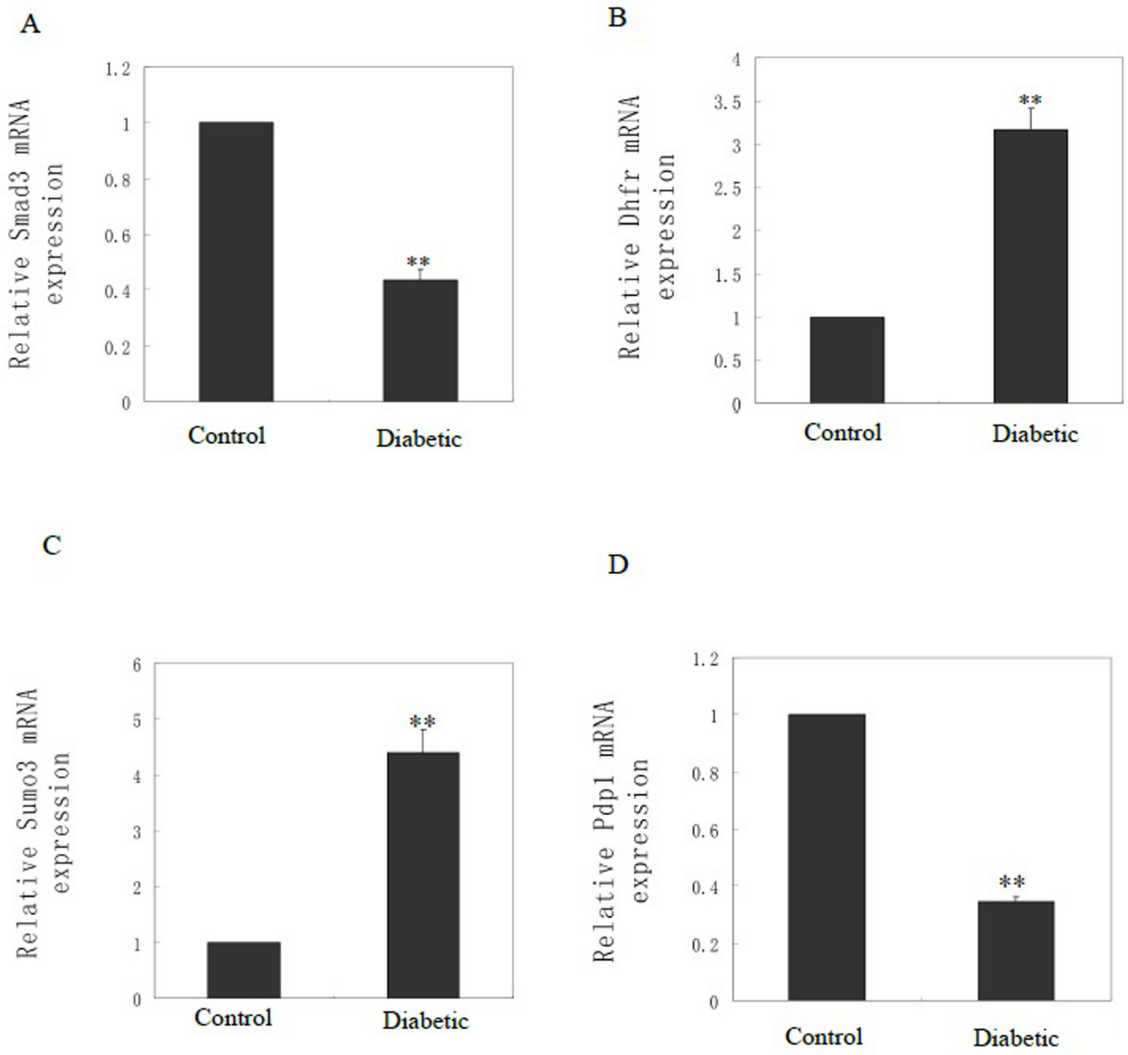
Genes with abnormal methylation in the upstream region differentially expressed in the diabetic and control groups. qRT-PCR was used to analyze mRNA expression of Smad3 (A), Dhfr (B), Sumo3 (C) and Pdp1 (D) in fetal heart samples from normal controls (n = 6) and diabetic (n = 6). β-actin was used as the internal reference. **P-value < 0.01.

The DNA methylation status of the upstream region is critical because of its role in regulating gene expression [20]. Two genes with abnormal CCGG methylation in the upstream region, *Smad3* [21] and *Dhfr* [22], were selected from the results of Methylation-RAD sequencing because of their previously established roles in CHD. qRT-PCR showed that the expression of *smad3* mRNA was decreased (Fig 5A) and the expression of *dhfr* mRNA was increased in the diabetic group (Fig 5B). We also examined the mRNA expression of several genes with abnormal CCNGG methylation in the upstream region under hyperglycemia using qRT-PCR. These genes were reported to be related to heart development and function. Specially, *sumo3* gene was reported to mediate cardiac development, metabolism and increased apoptosis and play a pathogenic role in the development of cardiomyopathy and heart failure [23, 24]. *pdp1* abnormal expression was associated with cardiomyocytes differentiation and cardiovascular disease [25, 26]. qRT-PCR showed that the expression of *sumo3* mRNA was increased (Fig 5C), and *pdp1* mRNA expression was decreased in the diabetic group (Fig 5D). These results suggested that abnormal methylation in the upstream region regulated gene expression under hyperglycemia.

### GO analysis of differential methylation sites

**Fig 6:**
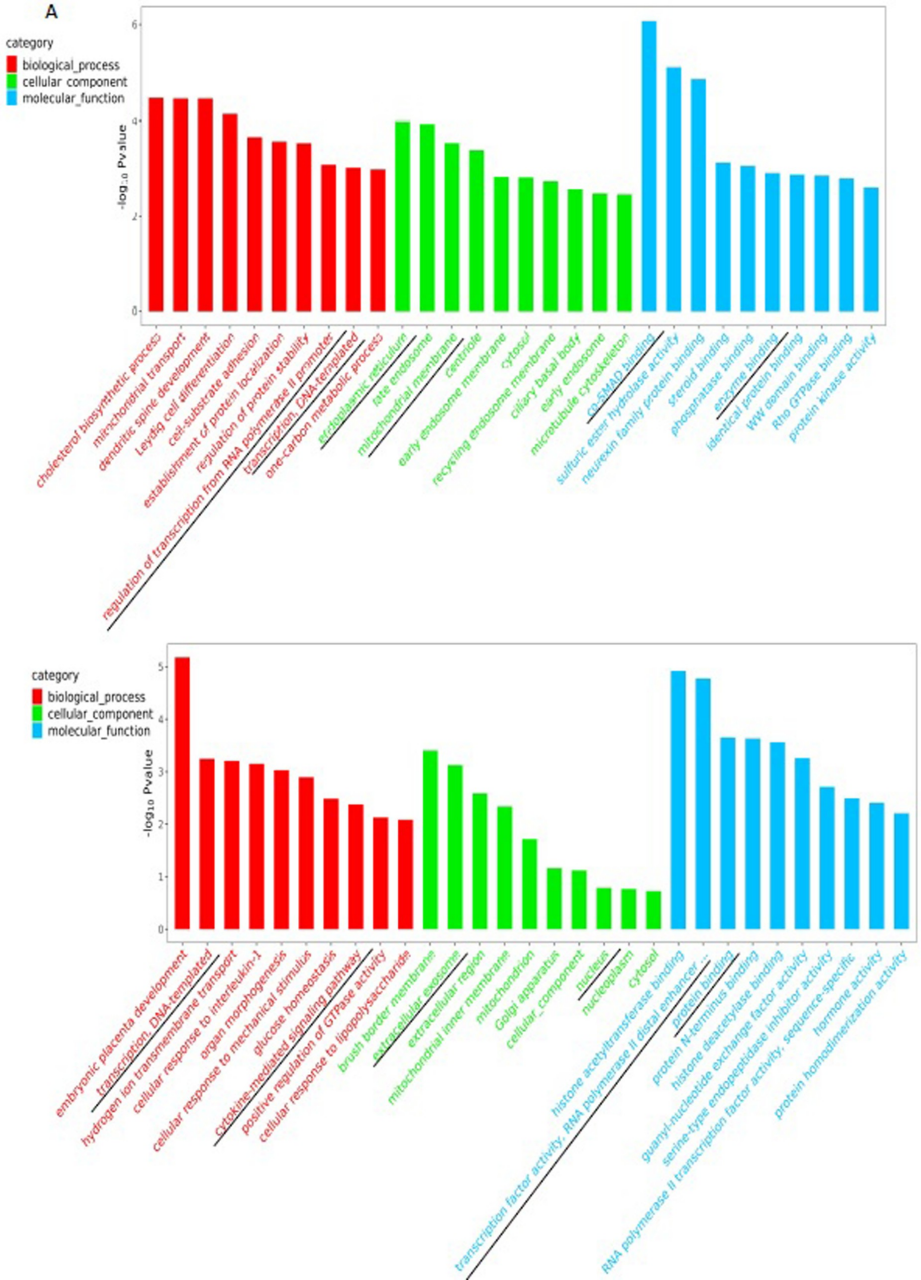
GO enrichment analysis of differentially methylated genes in the upstream region. (A) CCGG GO enrichment analysis; (B) CCNGG GO enrichment analysis, including cellular component, molecular function, and biological process. The representative results are marked with a black glide line.

We next examined the differentially expressed genes (DMGs) regulated by upstream methylation by GO enrichment analysis. The top 10 genes of every significant GO category with a *P-value* < 0.05 are listed in Fig 6. The CCGG-based DMGs involved in biological process were mainly related to the regulation of transcription from RNA polymerase II promoter and transcription, DNA-templated. The CCGG-based DMGs involved in the cellular component were mainly related to the endoplasmic reticulum and mitochondrial membrane. The CCGG-based DMGs involved in the molecular function were mainly related to co-SMAD binding and enzyme binding (Fig 6A and Table 2). The CCNGG-based DMGs in biological process were mainly related to transcription, DNA-templated and cytokine-mediated signaling pathways. The CCNGG-based DMGs involved in the cellular component were mainly related to extracellular exosome and nucleus. The CCNGG-based DMGs involved in the molecular function were mainly relative to transcription factor activity, RNA polymerase II distal enhancer sequence-specific binding, and protein binding (Fig 6B and Table 3).

**Table 2:** Diabetic-vs-control-CCGG.Diff.GO-classification.

Category	Term	Genes
biological_process	regulation of transcription from RNA polymerase II promoter	Zfp295; Cdx4; Smad3; Foxo4; Tbl1x; Tceal1; Etv4; Maged1; Tceal8; Zfp770; Zfp494; Ar; Brwd3; Pou3f4; Tceal3; Med14
	transcription, DNA-templated	Mga; Zfp449; Ctbp2; Smad3; Hes7; Smad6; Zcchc12; Hdac8; Foxo4; Pir; Nono; Tceal1; Tceal8; Zfp770; Zfp494; Rbbp7; Ar; Setd7; Pax7; Nr0b1
cellular_component	endoplasmic reticulum	Rnf128; Bcap31; Derl1; Emd; Gpat3; Pla2g4a; Erp29; Acsl4; Alg13; Ebp; Aldh3a1; Dnase1l1; Dhcr24; Slc35a2; Tor1b; Nkrf; Porcn; Tmem64; Pgrmc1; Zdhhc15; Prss50; Yipf6; Acsl1; Nsdhl; Zdhhc9; Prdx4
	mitochondrial membrane	Slc25a14; Acsl4; Slc25a30; Taz; Mt-nd6; Tmem126b
molecular_function	co-SMAD binding	Smad3; Smad6; Cited1; Usp9x
	enzyme binding	Smad3; Golph3; Cbx3; Msh3; Foxo4; Crb2; Rac1; Dhcr24; Kalrn; Ar; Snx12; Lamp2; Abcd1

**Table 3:** Diabetic-vs-control-CCNGG.Diff.GO-classification.

Category	Term	Genes
biological_process	transcription, DNA-templated	Col4a2; Cand1; Ablim2; Mapk3; Foxp3; Foxr1; Jdp2; Hif1a; Thrb; Rxrg
	cytokine-mediated signaling pathway	Sh2b2; Csf2rb; Rela
cellular_component	extracellular exosome	Col4a2; Csnk2b; Cand1; Tmem27; Galnt2; Sncg; Grid1; Slc5a5; Krt84; Spint1; Gmppb; Mapk3; Igfbp3; Slc9a3; Mmrn2; Rab33b; Mylk; Mt-atp6; Tpp1; Sumo3; Thrb; Chmp6; Mt-co2; Gpc4; Mid2
	nucleus	Csnk2b; Elac2; Cand1; Birc3; Slc5a5; Tbata; Ccne2; Ankrd2; Mapk3; Igfbp3; Foxp3; Habp4; Foxr1; Ppp1r16b; Prmt2; Jdp2; Clk3; Hif1a; Mycs; Tex24; Sumo3; Csnk2a2; Thrb; Rxrg; Cebpb; Dffb; Macrod1; Gpc4
molecular_function	transcription factor activity, RNA polymerase II distal enhancer sequence-specific binding	Foxp3; Hif1a; Cebpb; Rela
	protein binding	Ghrl; Fbxl20; Asb6; Dnaja4; Col4a2; Adgra3; Csnk2b; Gpank1; Sptssa; Cbln3; Nmu; Mt-nd3; Cand1; Tmem27; Galnt2; Sncg; Lats1; Birc3; Stx5; Tbata; Mt-cox3; Tusc2; Chrm4; Gmppb; Tnnt1; Fgfr4; Cldn8; Usp19; Ccne2; Pus7l; Ankrd2; Mapk3; Igfbp3; Foxp3; Mbl1; Habp4; Foxr1; Slc9a3; Kcnk16; Ppp1r16b; Abhd17a; Eif2b2; Mmrn2; Rab33b; Prmt2; Nrros; Jdp2; Clk3; Arhgef39; Hif1a; Mylk; Pdp1; Tpp1; Sumo3; Csnk2a2; Arhgef4; Sh2b2; Thrb; Chmp6; Cep104; Csf2rb; Rxrg; Cebpb; Dffb; Macrod1; Mt-co2; Il18r1; Mid2; Bend3; Epn2; Rela

### KEGG analysis of differential methylation sites

**Fig 7:**
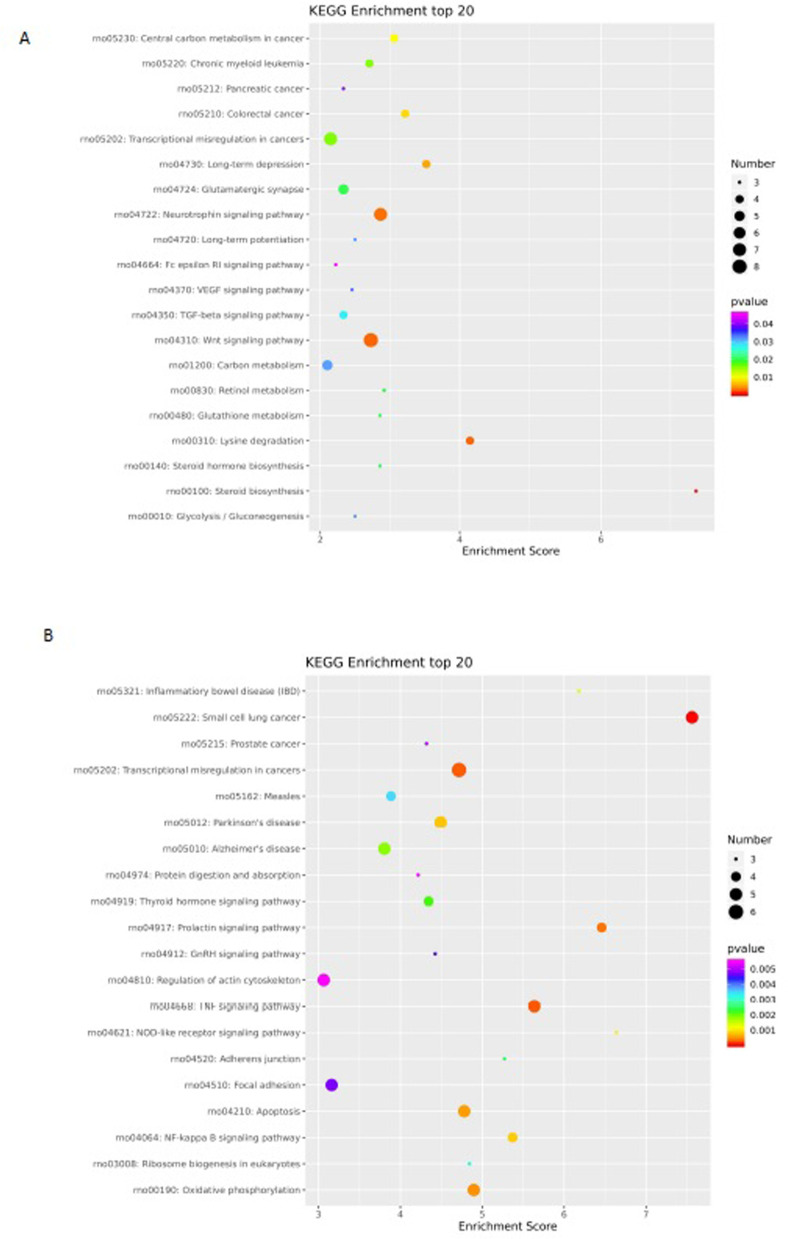
KEGG pathway classification of differentially methylated genes in the upstream region. KEGG pathway analysis showed that the differentially methylated genes were involved in different signaling pathways. (A) CCGG KEGG enrichment analysis; (B) CCNGG KEGG enrichment analysis.

We then performed KEGG analysis for the DMGs. As shown in Fig 7A and Table 4, the CCGG-based DMGs were mainly involved in the following biochemical signaling pathways: the Wnt signaling pathway, neurotrophin signaling pathway, transcriptional misregulation in cancers, TGF-β signaling pathway and VEGF signaling pathway. The CCNGG-based DMGs were involved in transcriptional misregulation in cancers, TNF signaling pathway, apoptosis, focal adhesion, NF-kB signaling pathway and Oxidative phosphorylation (Fig 7B and Table 5). These results suggest that these genes may play dominant roles in cardiomyocyte apoptosis and heart disease.

**Table 4:** Diabetic-vs-control-CCGG.Diff.KEGG-classification.

Classification	Gene number	Genes
Wnt signaling pathway	8	Ctbp2; Smad3; Tbl1x; Rac1; Nfatc4; Porcn; Plcb3; Gpc4
Neurotrophin signaling pathway	7	Mapk1; Rac1; Maged1; Shc3; Rps6ka3; Irak1; Ngfr
Transcriptional misregulation in cancers	7	Gria3; Golph3; Tfe3; Etv4; Pax7; AABR07018028.1; Ngfr
TGF-beta signaling pathway	4	Smad3; Mapk1; Smad6; Acvr2b
VEGF signaling pathway	3	Pla2g4a; Mapk1; Rac1
Glycolysis / Gluconeogenesis	3	Aldh3a1; Pgk1; Pdha1

**Table 5:** Diabetic-vs-control-CCNGG.Diff.KEGG-classification.

Classification	Gene number	Genes
Transcriptional misregulation in cancers	6	Birc3; Spint1; Igfbp3; Rxrg; Cebpb; Rela
TNF signaling pathway	5	Birc3; Mapk3; Cebpb; Il18r1; Rela
Apoptosis	5	Birc3; Mapk3; Csf2rb; Dffb; Rela
Focal adhesion	5	Col4a2; Birc3; Col9a2; Mapk3; Mylk
NF-kappa B signaling pathway	4	Csnk2b; Birc3; Csnk2a2; Rela
Oxidative phosphorylation	4	Mt-nd3; Mt-cox3; Mt-atp6; Mt-atp8; Mt-co2

## Discussion

Maternal pregestational diabetes is an arduous environment for embryonic development and results in an increased incidence of congenital heart malformations [1–3]. However, few studies have focused on the pathogenesis of congenital malformations under hyperglycemia. Hyperglycemic rat models were established and abnormal hearts were obtained in our previous studies [11, 12]. In this study, we investigated the epigenetic mechanism underlying maternal diabetes–induced CHD. We used MethyIRAD sequencing to screen new methylation sites and genes related to fetal heart malformation under hyperglycemia. We performed a comparative analysis of DNA methylation profiles of the fetal heart under hyperglycemia by MethyIRAD. Our data showed almost the entire genome with enough depth to identify differentially methylated regions with high accuracy. Our results suggest that MethyIRAD is a cost-effective approach for comprehensive analyses of genome-wide DNA methylation.

Previous studies have demonstrated that DNA methylation in different region plays different roles. For example, DNA methylation of the upstream region regulates gene expression [27], while DNA methylation of gene body regions modulates transcription elongation [28]. We analyzed the distribution of the methylated CCGG and CCNGG sites on the distinct genomic regions. Though the numbers of methylated CCNGG sites were lower than those of methylated CCGG sites (Fig 4A and 4B), the distributions of the methylated CCGG and CCNGG sites on distinct genomic regions were similar. Most of the examined methylation sites in the fetal heart were enriched in intergenic and intron regions. Few studies have examined the role of DNA methylation in these regions. Further research is needed to explore the functional consequence and regulatory implications of DNA methylation in intergenic and intron regions in the fetal heart.

The region 2 kb upstream of the TSS contains binding sites for transcription factors and plays an important role in regulating gene expression. Methylation of this region leads to decreased gene expression, while gene expression is increased when this region is unmethylated [27]. Our results showed that the expression levels of all four genes examined in the qRT-PCR analysis were consistent with the Methylation-RAD sequencing results. For example, increased CCGG methylation level in the upstream regulatory region of the Smad3 gene under hyperglycemia led to downregulation of the mRNA level of Smad3. Li et al. reported that the SNP rs2289263 in the SMAD3 gene is associated with ventricular septal defect in a Chinese Han population [21], and Lim et al. reported that Smad3 loss resulted in defects in heart development with failure of ventricular wall thickening [29]. Our results also showed an association of abnormal expression of Smad3 with heart development defects. Together these results suggest that Smad3 plays important roles in CHD development and that targeting its aberrant methylation may be a therapeutic strategy. In addition, the decrease of CCGG methylation level in the upstream regulatory region of the Dhfr gene caused increased mRNA level under hyperglycemia. Dhfr promoter methylation was previously associated with ischemic stroke [30]. However, the relationship between Dhfr methylation with CHD has not been reported. Our findings may thus reveal a new pathogenic mechanism of Dhfr in the development of CHD.

We also performed functional annotation of DMGs through GO and KEGG analysis to examine the potential processes and pathways. The results of functional analysis of CCGG-based DMGs by GO analysis were partially consistent with those by KEGG analysis. KEGG and GO analysis both showed that CCGG-based DMGs (such as Smad3 and Smad6 genes) were mainly involved in TGF-β (Table 4) and co-SMAD (Table 2) signaling pathways, respectively. These results suggested that abnormal changes of the TGF-β-SMAD signaling pathway may play an important role in CHD development. Therefore, further research should examine the expression and regulation of TGF-β pathway components and the potential association with CHD.

DMGs by the CCNGG sites were mainly related to the apoptotic signaling pathway. Our previous study showed that apoptotic cardiomyocytes were increased under the hyperglycemic environment [11, 12]. These genes with different methylation levels (such as Csf2rb, Dffb genes) may encode proteins involved in the regulation of apoptosis (Table 5). Sumo3 from GO assay has been reported to mediate cardiomyocyte apoptosis and myocardial infarction [25, 26]. However, the relationship between Csf2rb and Dffb and cardiomyocyte apoptosis and heart development has not been reported. Our next study will examine the potential function and mechanism of these apoptosis-regulating factors in CHD.

## Supporting information

S1 Table Primer pairs for β-actin, Smad3, Dhfr, Sumo3, Pdp1 Mt-atp6 were listed in S1 Table. (DOCX)

S1 Fig Morphological changes in heart tissue of embryos exposed to maternal diabetes. Ventricular myocardial walls were thinner in the diabetic embryos (right panel) compared with the non-diabetic embryos (left panel) at E15.5 by hematoxylin and eosin (H&E) staining. The arrow represents the abnormal heart development region.
(TIF)
