# Supplementary material for: Genome-wide analysis of methylation in rat fetal heart under hyperglycemia by methylation-dependent restriction site–associated DNA sequencing
Source: PLoS One. 2022 May 11;17(5):e0268117. doi: 10.1371/journal.pone.0268117 (PMC9094537; doi:10.1371/journal.pone.0268117)
Supplement: S1 Table — (DOCX) [file pone.0268117.s001.docx]

| Supplementary table1. primers for PCR | | |
| --- | --- | --- |
| Gene | Sense | Anti-sense |
| Smad3 | 5’- GCTTGGTGAAGAAGCTCAAGA-3’ | 5’-GCGTCCATGCTGTGGTTCAT-3’ |
| Dhfr | 5'-TTCTCCCAGAATACCCAGGC-3' | 5'-GCCACCCCTCCCTAAAGAAA-3' |
| Sumo3 | 5'-GCAAGCTGATGAAGGCCTAC-3' | 5'-TTGTGTGATTGGTGTGGCTG-3' |
| Pdp1 | 5'-GGCAGCTTGTTTCCTTACCC-3' | 5'-GATGGAAGGAGTCTGCAGGT-3' |
