# Supplementary figures and images for: Genome-wide analysis of methylation in rat fetal heart under hyperglycemia by methylation-dependent restriction site–associated DNA sequencing
Source: PLoS One. 2022 May 11;17(5):e0268117. doi: 10.1371/journal.pone.0268117 (PMC9094537; doi:10.1371/journal.pone.0268117)

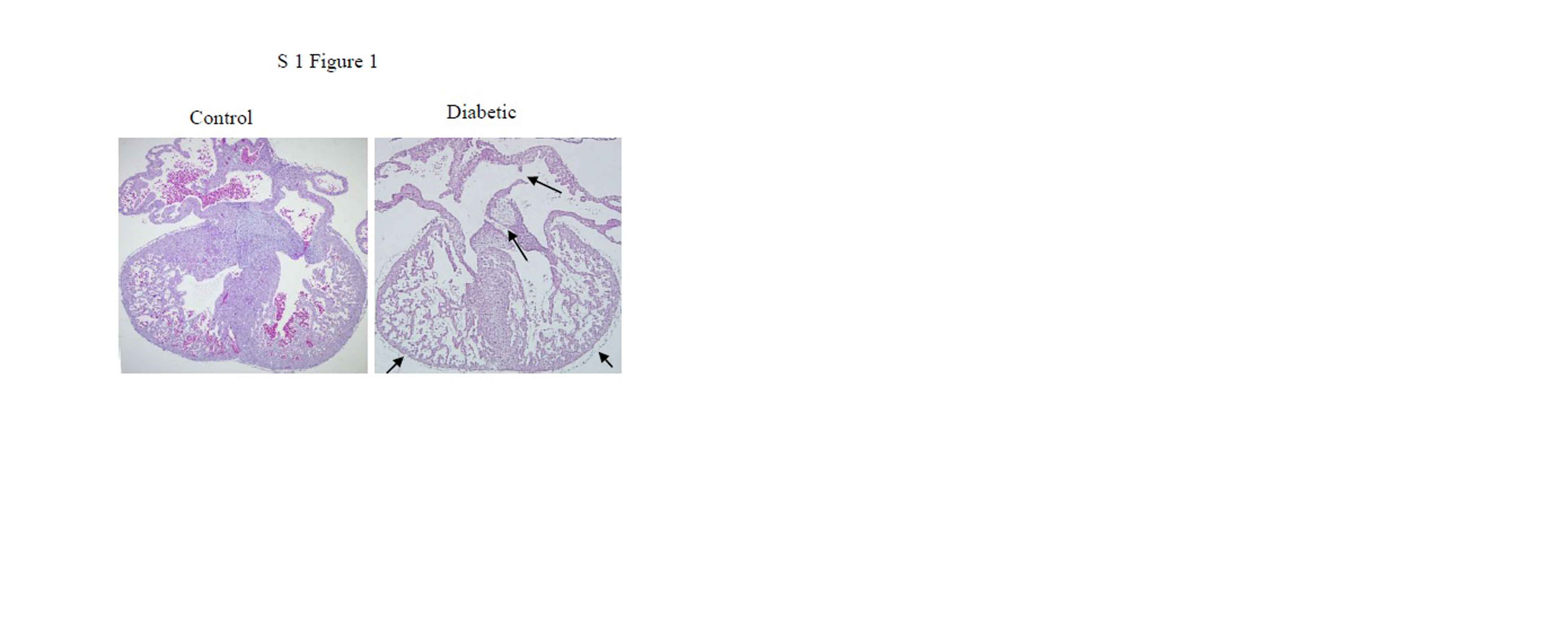

Supplement: S1 Fig — Ventricular myocardial walls were thinner in the diabetic embryos (right panel) compared with the non-diabetic embryos (left panel) at E15.5 by hematoxylin and eosin (H&E) staining. The arrow represents the abnormal heart development region. (TIF) [file pone.0268117.s002.tif]
